# Atimiaphobia: The Undiscovered Burden of Honor Cultures and Shame Societies on Mental Health—Development and Validation of Atimiaphobia Scale

**DOI:** 10.1002/pchj.70095

**Published:** 2026-05-04

**Authors:** Waqar Husain, Muhammad Ahmad Husain, Farrukh Ijaz, Khaled Trabelsi, Achraf Ammar, Haitham Jahrami

**Affiliations:** ^1^ Department of Humanities COMSATS University Islamabad, Islamabad Campus Islamabad Pakistan; ^2^ Research Laboratory Education, Motricité, Sport et Santé, EM2S, LR19JS01 High Institute of Sport and Physical Education of Sfax, University of Sfax Sfax Tunisia; ^3^ Department of Movement Sciences and Sports Training, School of Sport Science The University of Jordan Amman Jordan; ^4^ Department of Training and Movement Science Institute of Sport Science, Johannes Gutenberg‐University Mainz Mainz Germany; ^5^ Research Laboratory, Molecular Bases of Human Pathology, LR19ES13, Faculty of Medicine of Sfax University of Sfax, Sfax 3000 Sfax Tunisia; ^6^ Nutrition and Food Technology Department, Agriculture School The University of Jordan Amman Jordan; ^7^ Government Hospitals, Ministry of Health, Kingdom of Bahrain Manama Bahrain; ^8^ Department of Psychiatry, College of Medicine and Health Sciences Arabian Gulf University Manama Bahrain

**Keywords:** fear of losing honor, fear of shame, honor cultures, measurement and assessment, shame societies

## Abstract

The psychological impact of honor cultures and shame societies on the general population has not been examined through a diagnostic lens. Atimiaphobia is a newly recognized psychological condition characterized by an intense fear of losing honor or being labeled shameless, deeply rooted in honor cultures and shame societies. To assess this construct, the Atimiaphobia Scale (AtiPhoS) was developed and rigorously validated. The study was conducted in a series of four phases involving 1232 participants (M_age_ = 27 years; women = 48.9%). The validation of the AtiPhoS involved exploratory and confirmatory factor analyses along with convergent and predictive validity. The AtiPhoS, comprising 15 items (English) and four subscales (fear of being labeled shameless, fear of violating social norms, fear of public judgement, fear of losing self‐respect and honor) demonstrated excellent reliability (α = 0.824; ICC = 0.989). The model fit indices, such as CFI (0.933), TLI (0.916), RMSEA (0.065), and SRMSR (0.044), showed strong validity. Convergent validity was demonstrated by the scale's significantly positive correlation with the Experience of Shame Scale (*r* = 0.377) and the anxiety sub‐scale of the Depression, Anxiety, and Stress Scale (*r* = 0.262). The predictive validity of the AtiPhoS was established through its inverse predictive values for social intelligence (*β =* −0.229). A significant positive correlation was found between atimiaphobia and age. Women and married individuals exhibited significantly higher levels of atimiaphobia compared with men and unmarried individuals, respectively. The study provides compelling evidence that atimiaphobia is a distinct and measurable phenomenon, contributing to the broader understanding of cultural stressors related to honor and shame.

## 1 Introduction

Honor is a set of guiding codes that help individuals maintain a positive self and social image within a community (Rodriguez Mosquera 2016). It is based on self‐worth and social appraisal, where respect from others enhances self‐esteem, leading to feelings of pride, while dishonor evokes shame and rage (Olsthoorn 2005; Patterson 2019; van Osch et al. 2013). Honor functions within cultural frameworks, requiring adherence to social values to be maintained (Leung and Cohen 2011). It is a shared family asset that can be lost and is difficult to regain, necessitating swift responses to dishonor (Uskul and Cross 2019). The restoration of honor may involve extreme measures such as social isolation, psychological and physical abuse, forced suicide, forced marriage, marital rape, and even murder, as those who do not act against affronts are seen as weak and guilty (Christianson et al. 2021; Cross et al. 2013).

Honor is classified into morality‐based, family, masculine, and feminine honor, with different codes for men and women (Christianson et al. 2021; Lopez‐Zafra et al. 2020; Rodriguez Mosquera 2016). Women are expected to adhere more strictly to social values, suppress desires, and avoid vocalizing their sexuality or making independent decisions (D'Lima et al. 2020). Emotional responses also play a role in honor adherence, as individuals with a strong sense of honor react more intensely to insults (Mosquera et al. 2008; van Osch et al. 2013). Married individuals are more likely to support honor‐based norms (Gengler et al. 2021). Honor killings are linked to low socioeconomic status and are found even in Western societies (Dayan 2021; Kaya and Turan 2018; Shier and Shor 2016). Religion also influences honor culture (Cihangir 2013).

Honor is more valued in collectivistic cultures, where social identity depends on reputation, status, and fulfilling family responsibilities (Aslani et al. 2016; Uskul and Cross 2019). Individualistic cultures, however, emphasize personal standards over social opinions (Cross et al. 2013). The culture of honor historically emerged in societies where wealth was easily stolen and justice systems were weak, leading men to adopt toughness to prevent exploitation, while women sought honor through giving birth to sons (Uskul and Cross 2019). It persists in lawless societies where state protection is weak (Leung and Cohen 2011), with reputation maintenance as a central function (R. P. Brown et al. 2018). Non‐compliance with honor codes results in distrust, whereas those who restore honor gain social respect (Cross et al. 2014; Leung and Cohen 2011). However, honor culture weakens where effective authority replaces reputation‐based social order (Nowak et al. 2016). In contrast, dignity cultures emphasize intrinsic self‐worth that cannot be diminished by others (Leung and Cohen 2011).

Shame is an emotion and can be defined as a negative self‐evaluation or feeling bad about one's self (Barrett 2015). Considering one to be flawed, insufficient, unwanted, useless, inferior, worthless, can all be examples of projecting shame (Tangney et al. 2014; Yakeley 2018). Shame is a social phenomenon and it requires audience to feel shame (Mills and Kellington 2012). Shame resilience theory regards shame as a reaction to abstain one from the feelings to being caught (B. Brown 2006) or being disgraced (Buechler 2008). The self‐discrepancy theory regards shame as one's guilt on moral grounds (Dearing et al. 2011; Fromson 2006). Shame results from a process of self‐evaluation in which the individual is involved in evaluating his or her own worth or self‐esteem (Berkovski 2015). Shame can also be taken as a worry about the disclosure of any negative information about a person to the society (Sznycer et al. 2015). The disclosure of the negative information to a larger group of people may further cause social defame and devaluation (Sznycer et al. 2017). The intensity of shame, therefore, increases with the likelihood of devaluation (Robertson et al. 2018). Shame is culturally determined and varies from culture to culture (Boiger et al. 2013; Furukawa et al. 2012; Nash 2017; Thomas et al. 2020). Collectivistic cultures promote ‘shame societies’ where individuals are afraid of social defame. Individualistic cultures, on the other hand, promote ‘guilt societies’ where individuals are worried to be legally punished for their wrong doings (Yakeley 2018).

Shame destroys the positive self‐image of a person (de Hooge et al. 2010). Shame is regarded as an emotional trauma which also carries low self‐esteem and a sense of uselessness (Tangney 2004). The biophysical and psychosocial discomfort developed by shame is easily observable through behaviors such as flushing in the face or body, lowering down one's head, shaking, and avoiding eye contact (Dearing et al. 2011). Due to a mixture of emotional, physical, or interpersonal components, the feeling of shame becomes intolerable (B. Brown 2006). Shame is positively correlated with guilt (Treeby et al. 2016), aggression (Zhu et al. 2019), and several mental issues such as depression, generalized anxiety disorder, social anxiety disorder (Fergus et al. 2010), and post‐traumatic stress disorder (Corrigan 2004). Women sense humiliation much faster than men, and adolescents feel more shame than adults (Orth et al. 2010). Separated men and women were found to have higher levels of shame as compared with single or married individuals (Kõlves et al. 2011; Kotrotsiou et al. 2013). Financial wellbeing is inversely correlated with shame (Blea et al. 2021; Richardson et al. 2017).

In collectivistic cultures, honor and shame are not independent psychological phenomena but structurally interdependent forces that together constitute the moral architecture of social life. In collectivistic societies, individuals are integrated into strong, cohesive in‐groups from birth, and throughout their lives are expected to fulfill obligations, act properly, and promote others' goals (Bhagat and Hofstede 2002). Within this relational framework, honor functions as the group's recognition of a person's worth, a kind of public credit rating where everyone has a score. This is particularly salient in the cultures of Asia, Africa, and the Middle East Squarespace. Because individual identity is defined through one's standing within the group, honor and shame operate as two sides of the same coin: honor is the reward for meeting communal expectations, and shame is the penalty for failing them. In these collectivistic contexts, people are ashamed for not fulfilling group expectations and must seek to restore their honor before the community. This dyadic relationship means that neither concept can be meaningfully understood in isolation. Empirical research further confirms this link. Those with collectivistic tendencies tend to report more shame and possess more highly elaborated shame cognitions (Bedford 2004; Wong and Tsai 2007). The interdependence of honor and shame in collectivistic cultures is thus not incidental but structural. Shame signals a breach of the honor code, and the restoration of honor is the culturally prescribed remedy for shame, rendering the two emotionally and socially inseparable (Dunaetz 2021; Leung and Cohen 2011).

Despite extensive scholarship on honor cultures and shame societies (Dayan 2021; Kaya and Turan 2018; Shier and Shor 2016), the existing literature has primarily examined honor and shame related phenomena through sociological, anthropological, or affective lenses (Cross et al. 2013; Uskul and Cross 2019), with limited attention to their measurement as stable psychological fear‐based constructs at the individual level. Although several instruments assess related constructs such as shame, social anxiety, or fear of negative evaluation, none specifically capture the culturally embedded fear of losing honor or being labeled shameless. The absence of a dedicated psychometric measure limits empirical investigation into the intensity, structure, and psychosocial correlates of this fear across populations. To address this gap, the present study aimed to develop and validate the Atimiaphobia Scale (AtiPhoS), a self‐report instrument designed to assess individual differences in fear of losing honor and fear of being perceived as shameless. The study focuses on establishing the factorial structure, reliability, and construct validity of the scale, thereby providing a robust measurement tool for future research on honor‐ and shame‐related psychological processes.

## 2 Method

The present study was conducted in four consecutive phases. In the first phase, the AtiPhoS was developed, and its factorial structure was explored through exploratory factor analysis (EFA). The second phase involved confirmatory factor analysis (CFA) to validate the scale's factor structure. In the third phase, the convergent validity of the AtiPhoS was assessed by examining its relationship with anxiety and shame. Finally, the fourth phase evaluated the predictive validity of the AtiPhoS in relation to social intelligence.

### 2.1 Development of Atimiaphobia Scale

Atimiaphobia is conceptualized as a psychological condition characterized by an intense fear of losing honor or being labeled shameless, particularly within sociocultural contexts where honor, moral reputation, and social evaluation hold central importance (Husain 2025). The development of the AtiPhoS followed established guidelines for scale construction, emphasizing conceptual clarity, content relevance, and grounding in prior empirical and theoretical literature.

A comprehensive review of literature on honor cultures, shame societies, social reputation, moral evaluation, and fear‐based psychosocial processes was conducted to identify core indicators relevant to the construct (Aslani et al. 2016; Barrett 2015; Berkovski 2015; Brown 2006; Cihangir 2013; Cross et al. 2014; Husain 2025; Nowak et al. 2016; Yakeley 2018). This review revealed that fear of honor loss and fear of being perceived as shameless are consistently associated with heightened concern about moral standing, sensitivity to public judgment, adherence to social norms, and preoccupation with maintaining personal and familial reputation. These themes recur through psychological, sociological, and cross‐cultural research examining honor, shame, and reputation management, particularly in collectivistic and honor‐based societies.

The initial item pool of the AtiPhoS consisted of 20 items designed to assess individual differences in fear related to losing honor or being labeled shameless within honor cultures and shame societies. Item development was guided by a dimensional conceptualization of this fear, recognizing that concerns about honor and reputation may range from culturally normative sensitivity to more elevated and psychologically burdensome preoccupation. Accordingly, the items were constructed to capture variability in the intensity and salience of honor‐related fear rather than to imply diagnostic severity or clinical thresholds.

Given the sociocultural centrality of honor, moral standing, and reputation in many societies, the items systematically reflected cognitive, emotional, and behavioral responses associated with concerns about social and moral evaluation. Cognitive items assessed preoccupation with reputational judgment, such as “I worry about being labeled shameless” and “I constantly think about how others judge my moral conduct.” Emotional items captured distress associated with perceived threats to honor, for example, “The thought of being publicly devalued makes me feel deeply uneasy.” Behavioral items reflected heightened sensitivity to social norms and avoidance of reputational disapproval, such as “I feel distressed at the idea of failing to conform to social norms.” Together, these components were intended to provide a comprehensive yet non‐pathologizing assessment of fear related to honor loss.

Care was taken to ensure that the AtiPhoS does not merely assess general social anxiety or nonspecific interpersonal discomfort. Rather than focusing on fear of social interaction or negative evaluation in general, the items emphasize moral and reputational concerns that are culturally embedded and often extend beyond the individual self. For instance, items such as “I am afraid of losing my honor within my family” reflect the collectivistic dimensions of reputation, where personal conduct is intertwined with family and community standing. Similarly, items such as “I worry that public opinion determines my worth” capture heightened attention to socially mediated moral evaluation rather than situational social performance.

Importantly, the items were intentionally worded to remain applicable across a broad range of experiences, from relatively common concerns about social respectability to more elevated fear that may be associated with psychological strain. As such, the AtiPhoS is designed as a dimensional measure suitable for use in general population research, enabling empirical investigation of how fear of honor loss varies in intensity and relates to psychosocial outcomes without presuming clinical impairment or phobic pathology.

The initial 20 items of the AtiPhoS were evaluated for face validity by a panel of five expert psychologists with extensive experience in psychosocial and psychometric research. The experts were provided with a detailed briefing on the concept of atimiaphobia and the objective of the proposed scale. Following their assessment, the panel confirmed that the items were appropriately aligned with the construct of atimiaphobia. Interrater reliability analysis demonstrated substantial agreement among the raters, as indicated by Cohen's weighted kappa (0.741), Fleiss's kappa (0.736), and Krippendorff's alpha (0.737).

### 2.2 Participants

The current study involved a non‐clinical, general community sample comprising 1232 conveniently selected participants from Islamabad, Pakistan (men = 602, 48.9%; women = 630, 51.1%; unmarried = 773, 62.7%; married = 459, 37.3%; age = 18–64 years, M = 26.72, SD = 8.943; education = matriculation to doctorate, M = graduation). The first phase involved 400 participants (men = 210, 52.5%; women = 190, 47.5%; unmarried = 252, 63%; married = 148, 37%; age = 18–57 years, M = 27.45, SD = 10.378; education = matriculation to doctorate, M = graduation). The second phase involved 469 participants (men = 210, 44.8%; women = 259, 55.2%; unmarried = 226, 48.2%; married = 243, 51.8%; age = 18–57 years, M = 28.05, SD = 9.082; education = matriculation to doctorate, M = graduation). The third phase involved 164 participants (men = 83, 50.60%; women = 81, 49.4%; unmarried = 135, 82.3%; married = 29, 17.7%; age = 18–48 years, M = 23.77, SD = 3.841; education = matriculation to doctorate, M = graduation). The fourth phase involved 199 participants (men = 99, 49.7%; women = 100, 50.3%; unmarried = 160, 80.4%; married = 39, 19.6%; age = 18–64 years, M = 24.54, SD = 7.468; education = matriculation to doctorate, M = graduation).

A convenience sampling technique was utilized to recruit participants for all phases of the study. The researcher personally approached individuals during visits to various academic institutions, government offices, and private organizations. Participation was entirely voluntary, and informed consent was obtained from all participants before their inclusion. The eligibility criteria required individuals to (a) be at least 18 years old and (b) have sufficient proficiency in English to complete the questionnaires.

### 2.3 Sample Size Determination

The sample size for this research was determined based on established guidelines for EFA, CFA, and convergent/predictive validity assessment. For EFA, a minimum of five participants per item is recommended (Comrey and Lee 1992; Tabachnick et al. 2013). Given the initial pool of 20 items, the minimum required sample size was calculated as 100 participants. This requirement was exceeded, with phase 1 including 400 participants. CFA typically demands a larger sample size, with recommendations suggesting at least 10 participants per estimated parameter in the model (Kline 2023). The CFA model in this study comprised 15 items and four factors, resulting in 36 estimated parameters (15 factor loadings, 15 error variances, and 6 factor covariances). Consequently, the minimum required sample size for CFA was calculated as 360 participants. This criterion was surpassed, with phase two including 469 participants, ensuring sufficient statistical power to validate the factor structure. Convergent and predictive validity is usually assessed through correlation or regression analyses, requiring an adequate sample size to detect meaningful relationships between scale scores and relevant outcome variables. A multiple regression analysis with three to five predictor variables and a moderate effect size (f^2^ = 0.15) requires approximately 77 to 92 participants to achieve 80% power at an alpha level of 0.05 (Cohen 2013). However, to enhance the robustness and generalizability of the findings, researchers often aim for larger samples. The data collection in phases three (*n* = 164) and four (*n* = 199) followed this approach, ensuring that all studies met or exceeded the minimum required sample size.

### 2.4 Instruments

#### 2.4.1 Anxiety Subscale of the Depression, Anxiety, and Stress Scale

Atimiaphobia reflects a fear‐based psychological condition characterized by heightened concern over reputational threat and moral evaluation. This theoretically overlaps with anxiety‐related processes such as anticipatory worry, hypervigilance, and physiological arousal. Prior research indicates that chronic fear of social or moral threat is associated with elevated anxiety symptoms (Asmundson 2002; Kenardy et al. 1992; Panayiotou et al. 2017). Therefore, a positive association between AtiPhoS scores and anxiety, as measured by the DASS–Anxiety subscale, was hypothesized as further evidence of convergent validity. The Depression, Anxiety, and Stress Scale (DASS) (Lovibond and Lovibond 1995) is a self‐report inventory comprising 42 items divided into three subscales: depression, anxiety, and stress. It is a well‐established scale that has been widely utilized in numerous studies across the globe (Clara et al. 2001; Crawford and Henry 2003; Husain and Gulzar 2020). The Anxiety subscale of the Depression, Anxiety, and Stress Scale—DASS was used to establish the convergent validity of the AtiPhoS, as both constructs share overlapping features related to heightened fear and psychological distress. The DASS‐Anxiety subscale contains 14 items measuring autonomic arousal, skeletal muscle effects, situational anxiety, and subjective experience of anxious affect. Since anxiety plays a central role in atimiaphobic experiences, a significant positive correlation between the AtiPhoS and the DASS‐Anxiety subscale would provide evidence of the scale's convergent validity, demonstrating that it effectively measures a construct closely related to general anxiety. The DASS‐Anxiety subscale showed excellent reliability in the current study (Cronbach's alpha = 0.913).

#### 2.4.2 Experience of Shame Scale

Fear of losing honor or being labeled shameless is conceptually grounded in shame‐related self‐evaluation and concern about moral and social standing. Shame has been theorized as a core affective response to perceived violations of social and moral standards, particularly in honor cultures where reputation and moral worth are socially regulated (Mosquera et al. 2008; Sheikh and Janoff‐Bulman 2010). Accordingly, atimiaphobia was expected to show a positive association with dispositional shame, as measured by the Experience of Shame Scale, supporting convergent validity. The Experience of Shame Scale (ESS) (Andrews et al. 2002) is a psychological measure to assess the multidimensional experience of shame across personal, behavioral, and social domains. It consists of 25 items rated on a 5‐point Likert scale. The scale has been widely utilized in many studies, consistently demonstrating strong reliability and validity (Vizin et al. 2016). Given that atimiaphobia is fundamentally characterized by an excessive fear of moral disgrace and social dishonor, the ESS serves as an ideal measure for establishing the convergent validity of the Atimiaphobia Scale. A strong correlation between these two scales would support the claim that atimiaphobia is deeply intertwined with shame‐related cognitions and emotional distress, further validating the construct measurement of the AtiPhoS. The ESS demonstrated excellent reliability in the current study (Cronbach's alpha = 0.928).

#### 2.4.3 Efficient Social Intelligence Scale

Fear of losing honor or being labeled shameless is also expected to influence social functioning, particularly individuals' ability to navigate interpersonal situations effectively. Excessive concern with social judgment and moral evaluation may undermine flexible social reasoning, assertiveness, and adaptive interpersonal behavior (Mayer et al. 2015; Rudolph and Conley 2005). Consequently, higher levels of atimiaphobia were expected to predict lower social intelligence, as measured by the Efficient Social Intelligence Scale, providing evidence for the scale's predictive validity. The Efficient Social Intelligence Scale (ESIS) (Husain, Kamal, et al. 2025) comprises 9 items and 4 subscales: knowledge, efficacy, relations, and autonomy. It utilizes a 7‐point Likert scale for responses (strongly disagree to strongly agree). The scale was regarded highly reliable (Cronbach's alpha = 0.830 for the scale and ranging from 0.824 to 0.882 for the subscales; item‐scale and item‐total correlations were reported to be highly significant = *p* < 0.001) and valid (CFI = 0.990; TLI = 0.983; RMSEA = 0.045) (Husain, Kamal, et al. 2025). It was used to assess the predictive validity of the AtiPhoS, as social intelligence plays a crucial role in navigating interpersonal relationships, understanding social norms, and managing reputational concerns. Given that atimiaphobia is characterized by an intense fear of losing honor and being socially devalued, individuals with higher levels of atimiaphobia may exhibit heightened sensitivity to social cues, reputational threats, and interpersonal judgments. Establishing a predictive relationship between atimiaphobia and social intelligence helps validate the scale by demonstrating its ability to anticipate relevant social and psychological outcomes. The ESIS demonstrated strong reliability in the present study (Cronbach's alpha = 0.711).

### 2.5 Ethical Considerations

This study was conducted in strict adherence to established ethical guidelines, including the principles outlined in the 1964 Helsinki Declaration and its subsequent amendments, to safeguard the rights, dignity, and well‐being of all participants. Ethical approval was granted by the departmental ethics review committee at COMSATS University (Code: CUI‐ISB/HUM/ERC‐CPA/2024–028) prior to data collection. Participation was entirely voluntary, with informed consent obtained from all individuals before their inclusion. Participants were thoroughly briefed on the study's purpose, assured of the confidentiality of their responses, and informed of their right to withdraw at any stage without any repercussions. Anonymity was strictly maintained, and no personal or identifiable information was collected. The study employed no deceptive practices, and participants faced no foreseeable risks or harm. All data were securely stored and used exclusively for research purposes, ensuring strict privacy and confidentiality.

### 2.6 Analysis

The collected data were recorded and analyzed using the Statistical Package for Social Sciences (SPSS, version 26), Analysis of Moment Structures (AMOS, version 20), and Jeffreys' Amazing Statistics Program (JASP, version 0.19.0). Prior to conducting exploratory and confirmatory factor analyses, the dataset was screened to ensure data quality and compliance with statistical assumptions. The dataset was first examined for missing values, and no missing responses were identified across the items included in the analyses. Consequently, no imputation procedures were required. The data were also inspected for potential outliers. Univariate outliers were examined using standardized *z*‐scores, while multivariate outliers were evaluated using Mahalanobis distance at a significance threshold of *p* < 0.001. Although a small number of responses showed relatively extreme values, these cases were retained in the dataset to preserve the natural variability of participants' responses and to capture the full range of individual differences in fear related to honor loss. Assumptions relevant to factor analysis were also evaluated. The suitability of the data for factor analysis was assessed using the Kaiser–Meyer–Olkin (KMO) measure of sampling adequacy and Bartlett's test of sphericity. Item distributions were examined using skewness and kurtosis statistics to evaluate univariate normality, and the values fell within acceptable ranges for factor analytic procedures. Multicollinearity was assessed through inspection of the inter‐item correlation matrix to ensure that correlations did not exceed recommended thresholds. For confirmatory factor analysis, multivariate normality was evaluated through inspection of distributional indices and Mardia's coefficient. The results indicated that the data met the assumptions required for structural equation modeling using maximum likelihood estimation.

To evaluate the reliability and validity of the AtiPhoS, both EFA and CFA were performed. In the EFA, maximum likelihood was employed for extraction along with promax rotation. The EFA results were assessed using extraction values, Bartlett's test of sphericity (BTS), the Kaiser–Meyer–Olkin (KMO) measure of sampling adequacy, comparative fit index (CFI), Tucker–Lewis index (TLI), root mean square error of approximation (RMSEA), standardized root mean square residual (SRMR), and total variance explained.

For the CFA, the model was estimated using maximum likelihood, and factors were permitted to correlate. Model fit was evaluated through multiple fit indices, including the chi‐square test, CFI, TLI, RMSEA, SRMR, and the goodness‐of‐fit index (GFI). Additionally, the KMO test, BTS, the heterotrait–monotrait (HTMT) ratio, Cronbach's alpha, and McDonald's omega were computed to assess the scale's reliability and construct validity.

To complement the classical test theory approaches (EFA and CFA), a confirmatory multidimensional item response theory (MIRT) analysis was conducted using the graded response model (GRM), which is appropriate for ordered categorical (5‐point Likert) data. A 4‐dimensional confirmatory model was specified exactly matching the factor structure validated in CFA: F1 (items 1–5: fear of being labeled shameless), F2 (items 6–8: fear of violating social norms), F3 (items 9–11: fear of public judgement), and F4 (items 12–15: fear of losing self‐respect and honor). Parameters were estimated via the Expectation–Maximization (EM) algorithm with standard errors. Model fit was evaluated with the M_2_ statistic, CFI, TLI, RMSEA, and SRMSR. Item‐level fit was assessed with the S‐X^2^ statistic. Discrimination (a) and threshold (b_1_–b_4_) parameters, standardized loadings, and marginal reliability were examined. The analysis was performed in R (version 4.4.1) with the *mirt* package (Chalmers 2012).

To examine relationships between variables, Pearson's correlation coefficient was utilized. Simple regression analysis was conducted to assess the predictive validity of the AtiPhoS. Furthermore, an independent samples *t*‐test was performed to measure the differences in atimiphobia based on gender and marital status.

## 3 Results

The final AtiPhoS comprises 15 items (in English) and four distinct subscales: fear of being labeled shameless (items 1–5), fear of violating social norms (items 6–8), fear of public judgement (items 9–11), fear of losing self‐respect and honor (items 12–15). The response scale involves five points, that is, *strongly disagree* (scored 1), *disagree* (scored 2), *don't know* (scored 3), *agree* (scored 4), *strongly agree* (scored 5).

### 3.1 Reliability

The AtiPhoS demonstrated good levels of reliability in all four phases of the current study (Table 1; Cronbach's alpha ranged from 0.754 to 0.835 for the overall scale and 0.631 to 0.869 for the four subscales). The item‐total correlations (Table 2; ranging from 0.300 to 0.633 with *p* < 0.001; mean = 0.470) and item‐scale correlations (ranging from 0.621 to 0.864 with *p* < 0.001; mean = 0.766) of the AtiPhoS items demonstrated a high degree of internal consistency during the EFA. The test–retest reliability of the AtiPhoS after a two‐week interval with the same participants (*n* = 30) was excellent (interclass correlation type = ICC3,1; point estimate = 0.989; lower 95% CI = 0.978; upper 95% CI = 0.995). The point estimates for the interclass correlation for the four subscales of the AtiPhoS were also excellent (0.969, 0.983, 0.982, & 0.948).

**TABLE 1:** Descriptive statistics, reliability, and data accuracy (*n* = 1232).

Variable	Items	α	M	SD	%	Range		Skewness	Kurtosis
						Potential	Actual		
*Phase 1*									
Atimiaphobia	15	0.754	49.700	7.797	66.267	15–75	31–70	0.098	−0.389
F1	5	0.803	17.360	4.370	69.440	5–25	5–25	−0.427	−0.338
F2	3	0.766	9.180	2.758	61.200	3–15	3–15	−0.220	−0.430
F3	3	0.787	7.420	2.892	49.467	3–15	3–15	0.378	−0.541
F4	4	0.631	15.740	2.571	78.700	4–20	5–20	−0.655	0.648
*Phase 2*									
Atimiaphobia	15	0.824	53.891	8.934	71.855	15–75	27–75	−0.424	0.070
F1	5	0.813	18.689	4.356	74.755	5–25	5–25	−0.768	0.143
F2	3	0.789	10.260	2.699	68.401	3–15	3–15	−0.520	−0.149
F3	3	0.788	8.987	3.028	59.915	3–15	3–15	−0.040	−0.831
F4	4	0.765	15.955	3.069	79.776	4–20	4–20	−0.845	0.571
*Phase 3*									
Atimiaphobia	15	0.835	52.760	8.764	70.347	15–75	33–75	0.010	−0.428
F1	5	0.869	18.450	4.578	73.800	5–25	5–25	−0.874	0.362
F2	3	0.845	10.480	2.795	69.867	3–15	3–15	−0.435	−0.649
F3	3	0.842	7.910	3.043	52.733	3–15	3–15	0.317	−0.746
F4	4	0.715	15.920	2.581	79.600	4–20	8–20	−0.609	0.641
Anxiety	14	0.913	15.650	10.010	37.262	0–42	0–42	0.605	−0.337
Shame	25	0.928	50.680	14.506	50.680	25–100	26–94	0.544	−0.238
*Phase 4*									
Atimiaphobia	15	0.808	50.310	9.019	67.080	15–75	30–71	0.027	−0.401
F1	5	0.861	16.450	5.102	65.800	5–25	5–25	−0.204	−0.752
F2	3	0.733	9.790	2.459	65.267	3–15	4–15	−0.083	−0.509
F3	3	0.810	8.500	3.177	56.667	3–15	3–15	0.148	−0.759
F4	4	0.696	15.570	2.964	77.850	4–20	4–20	−1.251	2.901
Social Intelligence	9	0.711	47.770	7.257	75.825	9–63	26–63	−0.412	−0.011

*Note: n* = Number of participants; α = Cronbach's Alpha; M = Mean; SD = Standard Deviation. F1: fear of being labeled shameless; F2: fear of violating social norms; F3: fear of public judgement; F4: fear of losing self‐respect and honor. Phase 1: *n* = 400; men = 210, 52.5%; women = 190, 47.5%; unmarried = 252, 63%; married = 148, 37%; age = 18–57 years, M = 27.45, SD = 10.378; education level = matriculation to doctorate, M = graduation. Phase 2: *n* = 469; men = 210, 44.8%; women = 259, 55.2%; unmarried = 226, 48.2%; married = 243, 51.8%; age = 18–57 years, M = 28.05, SD = 9.082; education level = matriculation to doctorate, M = graduation. Phase 3: *n* = 164; men = 83, 50.60%; women = 81, 49.4%; unmarried = 135, 82.3%; married = 29, 17.7%; age = 18–48 years, M = 23.77, SD = 3.841; education level = matriculation to doctorate, M = graduation. Phase 4: *n* = 199; men = 99, 49.7%; women = 100, 50.3%; unmarried = 160, 80.4%; married = 39, 19.6%; age = 18–64 years, M = 24.54, SD = 7.468; education level = matriculation to doctorate, M = graduation. All phases: *n* = 1232; men = 602, 48.9%; women = 630, 51.1%; unmarried = 773, 62.7%; married = 459, 37.3%; age = 18–64 years, M = 26.72, SD = 8.943; education = matriculation to doctorate, M = graduation.

**TABLE 2:** Item‐total and item‐scale correlations (Phase 1; *n* = 400).

Item no.	Item‐total correlation	Item‐scale correlations			
		F1	F2	F3	F4
1	0.568***	0.782***	0.033	0.205***	0.128*
2	0.480***	0.702***	0.121*	0.034	0.094
3	0.585***	0.744***	0.113*	0.137**	0.234***
4	0.633***	0.833***	0.202***	0.173***	0.094
5	0.550***	0.681***	0.034	0.196***	0.254***
6	0.440***	0.143**	0.851***	0.084	0.082
7	0.428***	0.099*	0.861***	0.162**	0.022
8	0.400***	0.093	0.763***	0.139**	0.080
9	0.516***	0.173***	0.171***	0.864***	0.114*
10	0.512***	0.147**	0.189***	0.864***	0.129**
11	0.476***	0.173***	0.047	0.798***	0.202***
12	0.408***	0.133**	0.109*	0.153**	0.724***
13	0.300***	0.064	−0.041	0.145**	0.682***
14	0.451***	0.217***	0.071	0.172***	0.730***
15	0.316***	0.162**	0.062	−0.005	0.621***

*Note:* F1: fear of being labeled shameless; F2: fear of violating social norms; F3: fear of public judgment; F4: fear of losing self‐respect and honor. *p < 0.05, **p < 0.01, ***p < 0.001; Factor structure is bold.

### 3.2 Exploratory Factor Analysis (EFA)

EFA was conducted to examine the underlying factor structure of the initial item pool of the AtiPhoS. The number of factors was not predetermined; rather, the factor structure was allowed to emerge from the data. Factor extraction was guided by commonly recommended criteria, including eigenvalues greater than 1.0, inspection of the scree plot, and the interpretability of the resulting factor structure. In the EFA (Table 2), maximum likelihood with promax rotation was applied to enhance interpretability. The sampling adequacy was adequate (Table 3; *n* = 400; KMO = 0.733 for the overall scale, which ranged from 0.657 to 0.831 for individual items). The adequacy of correlations between items was highly significant (Table 3; BTS: χ^2^ = 1812.320, df = 51, *p* < 0.001). During the EFA process, items were evaluated based on their factor loadings and cross‐loadings. Items with low loadings or substantial cross‐loadings across multiple factors were considered for removal in order to improve the clarity and interpretability of the factor structure. Following this iterative evaluation, five items were discarded during the exploratory factor analysis for not having the required thresholds for validity, such as communalities < 0.4 or cross loadings between factors above 0.2. (Osborne et al. 2008). The final AtiPhoS comprises 15 items and four distinct subscales. The factor loadings of these items ranged from 0.484 to 0.924 (Table 3). Several model fit indices, such as CFI (0.961), TLI (0.920), RMSEA (0.057), and SRMR (0.029), have strong validity.

**TABLE 3:** Exploratory factor analysis (Phase 1; *n* = 400).

Item no.	Item	Factor structure			
		F1	F2	F3	F4
1	I have an intense fear of being labeled shameless.	**0.823**	−0.098	0.071	−0.136
2	I am deeply afraid of losing my honor within my family.	**0.568**	0.056	−0.083	−0.032
3	I feel extremely anxious about being socially disrespected.	**0.538**	0.016	−0.006	0.176
4	I constantly worry that others will perceive me as shameless.	**0.924**	0.081	0.024	−0.197
5	The thought of being publicly devalued fills me with fear.	**0.484**	−0.074	0.072	0.201
6	I feel distressed at the idea of failing to conform to social norms.	0.036	**0.795**	−0.037	0.028
7	Breaking social norms and values makes me feel extremely anxious.	−0.001	**0.769**	0.081	−0.062
8	I fear that failing to comply with social expectations will bring dishonor upon me.	−0.020	**0.602**	0.020	0.070
9	I am terrified that others' opinions will damage my reputation.	0.013	0.067	**0.855**	−0.080
10	I constantly worry that public opinion will determine my worth.	−0.037	0.089	**0.874**	−0.053
11	I obsess over what others say about me, fearing it may harm my honor.	0.041	−0.071	**0.548**	0.109
12	I am consumed by the fear of losing my social reputation.	−0.067	0.076	0.038	**0.605**
13	The thought of appearing inferior in front of others makes me extremely uneasy.	−0.091	−0.082	0.060	**0.531**
14	I find the idea of being dishonored unbearable and distressing.	0.030	0.022	0.046	**0.556**
15	I live in fear of losing my self‐respect in front of others.	0.058	0.038	−0.152	**0.508**

*Note:* Factor structure is Bold. F1: fear of being labeled shameless; F2: fear of violating social norms; F3: fear of public judgement; F4: fear of losing self‐respect and honor. Extraction: Maximum likelihood with Promax rotation. Bartlett's Test of Sphericity: χ^2^ = 1812.320, df = 51, *p* < 0.001. Kaiser‐Meyer‐Olkin Measure of Sampling Adequacy: overall value = 0.733, values for individual items ranged from 0.657 to 0.831. Total variance explained: 0.479; Comparative Fit Index = 0.961; Tucker‐Lewis Index = 0.920; Root mean square error of approximation = 0.057; Standardized root mean square residual = 0.029. Sub‐scales: fear of being labeled shameless (items 1–5), fear of violating social norms (items 6–8), fear of public judgement (items 9–11), fear of losing self‐respect and honor (items 12–15). Response sheet: *strongly disagree* (scored 1), *disagree* (scored 2), *don't know* (scored 3), *agree* (scored 4), *strongly agree* (scored 5).

### 3.3 Confirmatory Factor Analysis (CFA)

**FIGURE 1:**
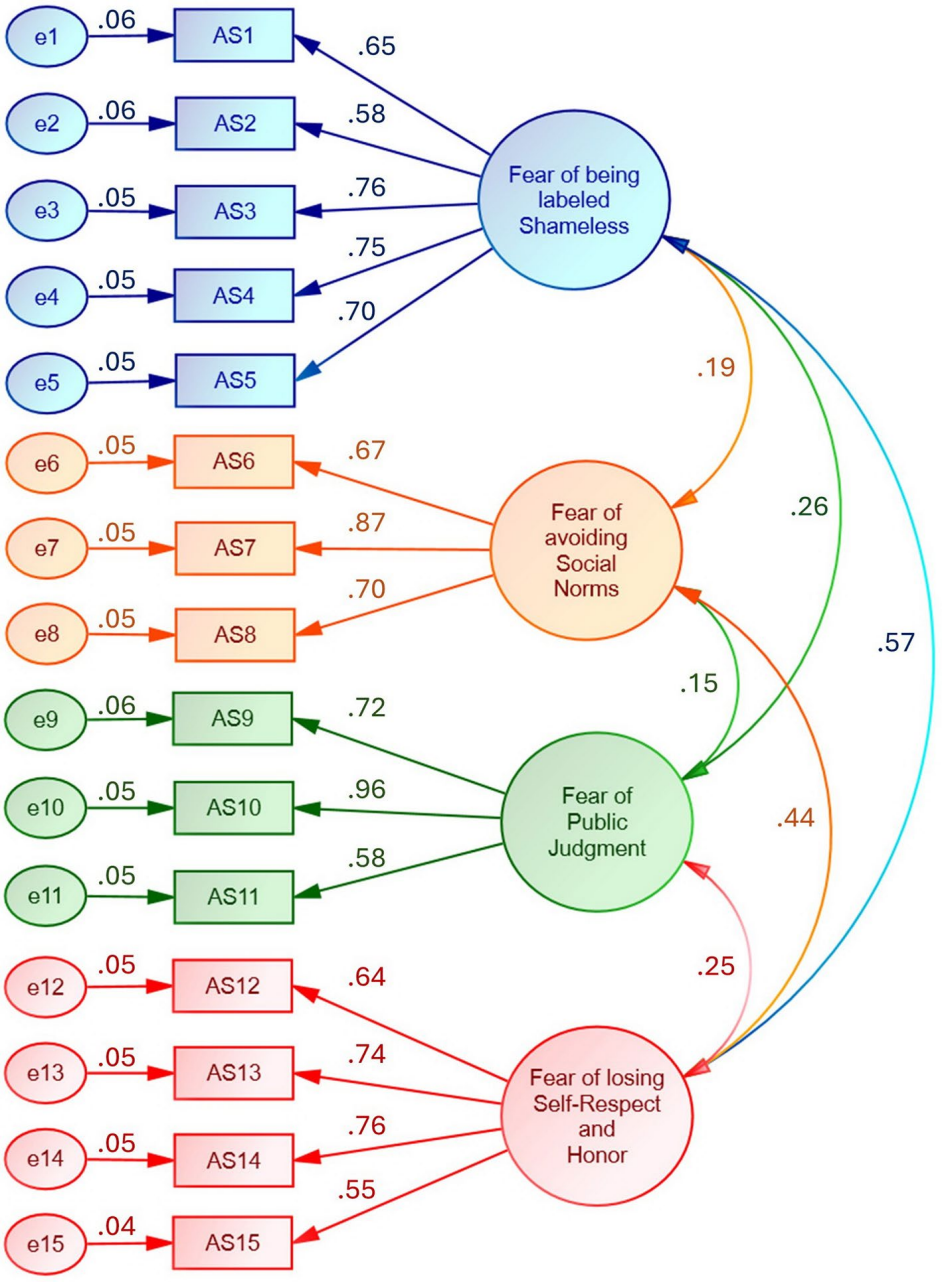
Confirmatory factor analysis (Phase 2; *n* = 469).

**FIGURE 2:**
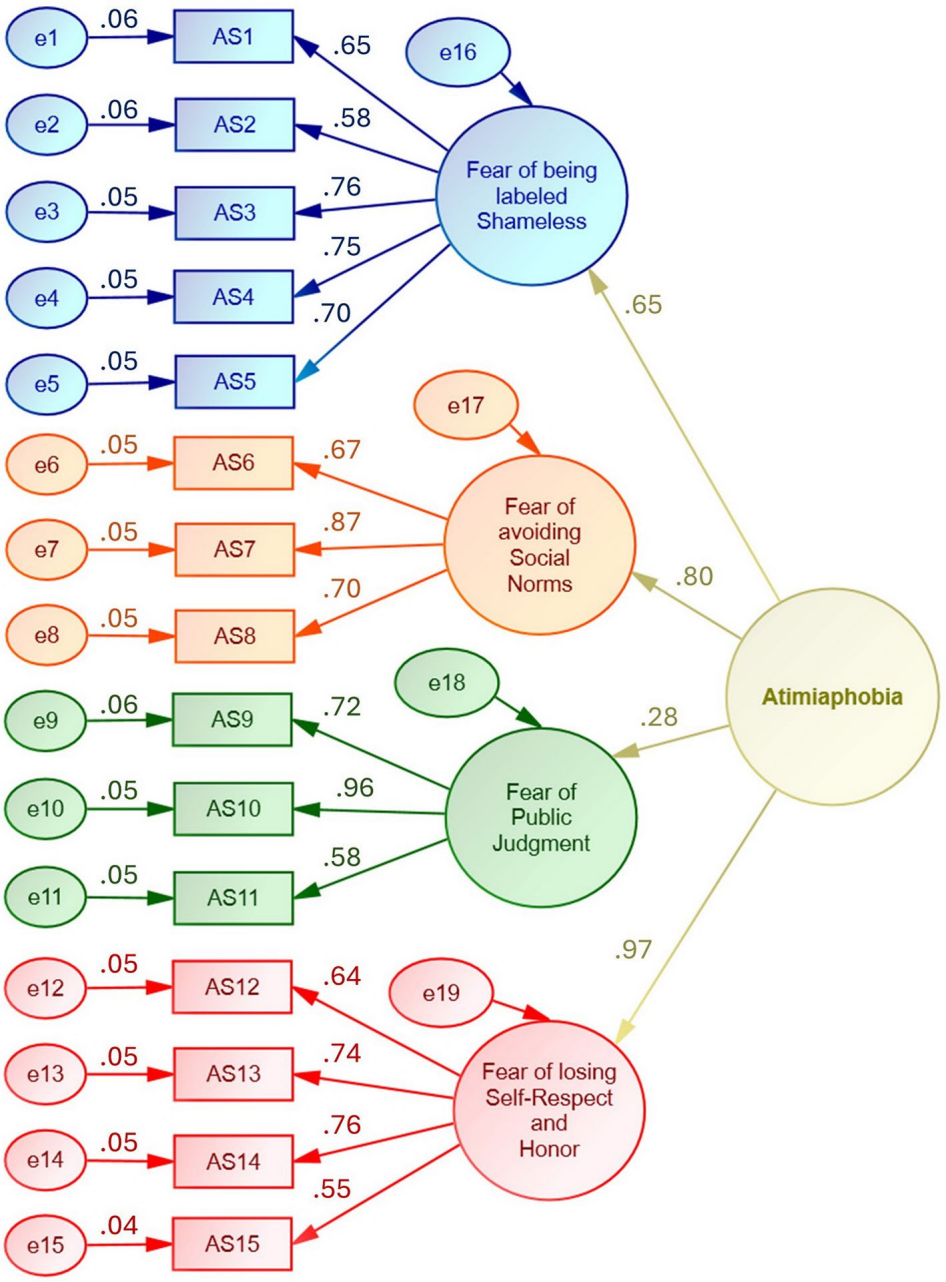
Second‐order factor analysis (Phase 2; *n* = 469).

The CFA was conducted on 15 items to test a 4‐factor model (Figure 1). The maximum‐likelihood extraction technique was utilized without rotation. The sampling adequacy was notable (Table 4; *n* = 469; KMO = 0.795 for the overall scale, which ranged from 0.651 to 0.870 for individual items). The adequacy of correlations between items was highly significant (Table 4; BTS: χ^2^ = 2528.435, df = 105, *p* < 0.001). The factor loadings were statistically significant (*p* < 0.001) and ranged from 0.553 to 0.960 (Table 4), indicating that the items were strongly related to the underlying factor. The average variances extracted for the four factors ranged from 0.474 to 0.592, which demonstrated adequate convergence. The reliability was also good (Coefficient ω = 0.861 for the scale and 0.815, 0.802, 0.810, & 0.780 for the 4 sub‐scales; Coefficient α = 0.824 for the scale and 0.813, 0.789, 0.788, & 0.765 for the 4 sub‐scales). The CFA model demonstrated good fit according to several fit indices, such as CFI (0.933), TLI (0.916), GFI (0.991), RMSEA (0.065), SRMR (0.044), and Heterotrait‐monotrait ratio (1). A higher‐order factor analysis (Figure 2) revealed that the fear of losing self‐respect and honor was the most influential aspect of atimiaphobia as compared to the fear of violating social norms, the fear of being labeled shameless, and the fear of public judgment.

**TABLE 4:** Confirmatory factor analysis (Phase 2; *n* = 469).

Factor	Item	Factor loadings				Residual variances			
		Estimate	SE	*z*	*p*	Estimate	SE	*z*	*p*
1	AS1	0.651	0.058	14.410	< 0.001	0.576	0.075	12.791	< 0.001
	AS2	0.584	0.055	12.747	< 0.001	0.659	0.070	13.738	< 0.001
	AS3	0.760	0.046	17.776	< 0.001	0.422	0.044	10.953	< 0.001
	AS4	0.749	0.048	17.400	< 0.001	0.439	0.049	11.217	< 0.001
	AS5	0.698	0.047	15.855	< 0.001	0.513	0.047	12.228	< 0.001
2	AS6	0.673	0.047	14.957	< 0.001	0.547	0.047	12.363	< 0.001
	AS7	0.868	0.050	19.651	< 0.001	0.247	0.060	5.341	< 0.001
	AS8	0.703	0.047	15.467	< 0.001	0.506	0.048	11.316	< 0.001
3	AS9	0.723	0.055	16.197	< 0.001	0.477	0.065	11.121	< 0.001
	AS10	0.960	0.052	21.792	< 0.001	0.079	0.077	1.453	0.146
	AS11	0.581	0.054	12.708	< 0.001	0.662	0.068	13.804	< 0.001
4	AS12	0.641	0.047	13.829	< 0.001	0.589	0.049	12.468	< 0.001
	AS13	0.735	0.048	16.500	< 0.001	0.460	0.050	10.747	< 0.001
	AS14	0.761	0.046	17.082	< 0.001	0.421	0.046	9.814	< 0.001
	AS15	0.553	0.041	11.561	< 0.001	0.694	0.038	13.506	< 0.001

*Note:* Extraction was performed using the Maximum‐likelihood extraction technique with no rotation. Additional Fit Measures: Comparative Fit Index (CFI): 0.933; Tucker‐Lewis Index (TLI): 0.916; Bentler‐Bonett Non‐normed Fit Index (NNFI): 0.916; Bentler‐Bonett Normed Fit Index (NFI): 0.903; Parsimony Normed Fit Index (PNFI): 0.723; Bollen's Relative Fit Index (RFI): 0.879; Bollen's Incremental Fit Index (IFI): 0.934; Relative Noncentrality Index (RNI): 0.933; Information Criteria: Log‐likelihood: −9481.045, Number of free parameters: 51, Akaike (AIC): 19064.091, Bayesian (BIC): 19275.772, Sample‐size adjusted Bayesian (SSABIC): 19113.908; Root mean square error of approximation (RMSEA): 0.065; RMSEA 90% CI lower bound: 0.055; RMSEA 90% CI upper bound: 0.074; RMSEA *p*‐value: 0.005; Standardized root mean square residual (SRMR): 0.044; Hoelter's critical N (α = 0.05): 201.841; Hoelter's critical N (α = 0.01): 221.967; Goodness of fit index (GFI): 0.991; McDonald fit index (MFI): 0.839; Expected cross‐validation index (ECVI): 0.747; Kaiser‐Meyer‐Olkin (KMO) Test: Overall KMO: 0.795; KMO for individual indicators ranged from 0.651 to 0.870; Bartlett's Test of Sphericity: χ^2^ = 2528.435, df = 105, *p* < 0.001; R‐Squared: Explained variance (*R* ^2^) for the items ranged from 0.306 to 0.921; Average variance extracted for 4 factors: 0.469, 0.579, 0.592, and 0.474; Heterotrait‐monotrait ratio = 1; Coefficient ω = 0.861 for the scale and 0.815, 0.802, 0.810, and 0.780 for the 4 sub‐scales; Coefficient α = 0.824 for the scale and 0.813, 0.789, 0.788, and 0.765 for the 4 sub‐scales.

### 3.4 Convergent and Predictive Validity

Strong convergent validity was demonstrated (Table 5) by the scale's strong correlation with the Experience of Shame Scale (*r* = 0.377, *p* < 0.001) and the anxiety sub‐scale of the Depression, Anxiety, and Stress Scale (*r* = 0.262, *p* < 0.001). The predictive validity of the AtiPhoS was established through its strong inverse predictive values for social intelligence (*R* = 0.229; *R* ^2^ = 0.052; Adj. *R* ^2^ = 0.047; df = 198; *F* = 10.874; *B* = −0.184; SE *B* = 0.056; *β* = −0.229; *t* = 92.857; *p* < 0.001).

**TABLE 5:** Correlations.

	Atimiaphobia	Fear of being labeled shameless	Fear of violating social norms	Fear of public judgement	Fear of losing self‐respect and honor
Shame	0.377***	0.380***	−0.006	0.247***	0.320***
Anxiety	0.262***	0.346***	−0.050	0.051	0.271***
Social Intelligence	−0.229**	−0.220**	0.093	−0.252***	−0.125
Age	0.231***	0.183***	0.130***	0.139***	0.141***
Education level	0.055	−0.007	0.107***	0.076***	−0.006

*Note:* **p < 0.01, ***p < 0.001.

### 3.5 Multidimensional Item Response Theory (MIRT) Analysis

The 4‐factor GRM (Table 6) provided good overall fit to the data (M_2_(45) = 240.00, *p* < 0.001, CFI = 0.943, TLI = 0.924, RMSEA = 0.059, SRMSR = 0.144). All items loaded strongly and exclusively on their hypothesized factors (standardized loadings 0.625–0.888; see summary output). Discrimination parameters were high (a = 1.36–3.28), indicating excellent ability to differentiate levels of atimiaphobia. Thresholds were well‐spread across the latent trait continuum. Most items showed acceptable fit (S‐X^2^ *p* > 0.05); only item 12 demonstrated poor fit (*p* < 0.001) and may warrant future review. Marginal reliability estimates for the four factors were strong (0.82–0.89). The orthogonal model (as specified) yielded zero factor correlations, consistent with the confirmatory structure. EAP factor scores were computed for all 1232 participants and saved for subsequent analyses.

**TABLE 6:** Item Parameter Estimates from the 4‐Factor Graded Response Model (*N* = 1232).

Item	Factor	Discrimination (a)	Threshold b_1_	b_2_	b_3_	b_4_	S‐X^2^ *p*‐value
1	F1	1.609	3.355	1.499	0.498	−2.041	0.028
2	F1	1.372	3.622	1.825	1.089	−1.074	0.084
3	F1	2.048	5.274	2.814	1.695	−1.594	0.083
4	F1	2.413	5.172	2.711	1.261	−2.171	0.046
5	F1	1.725	4.912	2.489	1.407	−1.641	0.009
6	F2	2.053	4.704	2.074	−0.104	−3.259	0.387
7	F2	2.298	4.402	1.610	0.091	−3.189	0.011
8	F2	1.685	4.541	1.951	0.015	−3.158	0.063
9	F3	2.441	2.500	−0.553	−1.874	−5.147	0.457
10	F3	3.282	3.194	−0.635	−2.480	−6.610	0.054
11	F3	1.364	2.344	0.307	−0.514	−3.247	0.068
12	F4	1.733	4.747	2.900	1.510	−1.632	< 0.001
13	F4	1.668	4.379	2.443	1.157	−1.912	0.012
14	F4	1.908	4.761	2.757	1.450	−1.449	0.206
15	F4	1.704	5.269	4.105	3.219	−0.193	0.110

*Note:* a = discrimination parameter (higher values indicate stronger differentiation); b_1_–b_4_ = category threshold parameters (location on the latent trait where probability of endorsing higher categories increases); S‐X^2^ = item‐fit statistic (*p* > 0.05 indicates acceptable fit). Factor loadings (standardized) ranged from 0.625 to 0.888. Model estimated with EM algorithm in *mirt* (Chalmers 2012). Item 12 showed misfit and may benefit from revision in future studies.

### 3.6 Age, Education, Gender, and Marital Status

Age (Table 5) exhibited a significant positive correlation with atimiaphobia (*r =* 0.231; *p* < 0.001) and all its counterparts: the fear of being labeled shameless (*r =* 0.183; *p* < 0.001), the fear of violating social norms (*r =* 0.130; *p* < 0.001), the fear of public judgment (*r =* 0.139; *p* < 0.001), and the fear of losing self‐respect and honor (*r =* 0.141; *p* < 0.001). No significant correlation was found between education and the overall levels of atimiaphobia (Table 5). Nonetheless, education showed a significant positive correlation with two specific dimensions of atimiaphobia: the fear of violating social norms (*r =* 0.107; *p* < 0.001) and the fear of public judgment (*r =* 0.076; *p* < 0.001).

Women projected significantly higher levels of atimiaphobia (M = 56.031, SD = 7.254, % = 80.044 vs. M = 47.378, SD = 8.003, % = 67.683; *p* < 0.001; Cohen's d = 1.134) and all its counterparts: the fear of being labeled shameless (M = 19.931, SD = 3.728, % = 79.724 vs. M = 15.702, SD = 4.412, % = 62.808; *p* < 0.001; Cohen's d = 1.037), the fear of violating social norms (M = 10.467, SD = 2.586, % = 69.780 vs. M = 9.231, SD = 2.755, % = 61.540; *p* < 0.001; Cohen's d = 0.463), the fear of public judgment (M = 9.027, SD = 2.948, % = 60.180 vs. M = 7.450, SD = 3.015, % = 49.667; *p* < 0.001; Cohen's d = 0.529), and the fear of losing self‐respect and honor (M = 16.606, SD = 2.647, % = 83.030 vs. M = 14.995, SD = 2.793, % = 74.975; *p* < 0.001; Cohen's d = 0.593) as compared to men (Table 7).

**TABLE 7:** Differences in atimiaphobia and its counterparts among men and women (*n* = 1232).

Variables	Men (*n* = 602)			Women (*n* = 630)			*t*(*1230*)	*p*	Cohen's *d*
	M	SD	%	M	SD	%			
Atimiaphobia	47.378	8.003	67.683	56.031	7.254	80.044	19.900	0.000	1.134
Fear of being labeled shameless	15.702	4.412	62.808	19.931	3.728	79.724	18.201	0.000	1.037
Fear of violating social norms	9.231	2.755	61.540	10.467	2.586	69.780	8.123	0.000	0.463
Fear of public judgement	7.450	3.015	49.667	9.027	2.948	60.180	9.281	0.000	0.529
Fear of losing self‐respect and honor	14.995	2.793	74.975	16.606	2.647	83.030	10.397	0.000	0.593

Married individuals projected significantly higher levels of atimiaphobia (M = 54.158, SD = 8.274, % = 72.211 vs. M = 50.405, SD = 8.759, % = 67.207; *p* < 0.001; Cohen's d = 0.437) and all its counterparts: the fear of being labeled shameless (M = 18.825, SD = 4.384, % = 75.300 vs. M = 17.295, SD = 4.619, % = 69.180; *p* < 0.001; Cohen's d = 0.338), the fear of violating social norms (M = 10.250, SD = 2.606, % = 68.333 vs. M = 9.633, SD = 2.792, % = 64.220; *p* < 0.001; Cohen's d = 0.226), the fear of public judgment (M = 8.817, SD = 2.965, % = 58.780 vs. M = 7.924, SD = 3.104, % = 52.827; *p* < 0.001; Cohen's d = 0.292), and the fear of losing self‐respect and honor (M = 16.266, SD = 2.734, % = 81.330 vs. M = 15.553, SD = 2.863, % = 77.765; *p* < 0.001; Cohen's d = 0.253) as compared to the unmarried (Table 8).

**TABLE 8:** Differences in atimiaphobia and its counterparts among married and unmarried (*n* = 1232).

Variables	Married (*n* = 459)			Unmarried (*n* = 773)			*t*(*1230*)	*p*	Cohen's *d*
	M	SD	%	M	SD	%			
Atimiaphobia	54.158	8.274	72.211	50.405	8.759	67.207	7.421	0.000	0.437
Fear of being labeled shameless	18.825	4.384	75.300	17.295	4.619	69.180	5.729	0.000	0.338
Fear of violating social norms	10.250	2.606	68.333	9.633	2.792	64.220	3.842	0.000	0.226
Fear of public judgement	8.817	2.965	58.780	7.924	3.104	52.827	4.963	0.000	0.292
Fear of losing self‐respect and honor	16.266	2.734	81.330	15.553	2.863	77.765	4.298	0.000	0.253

## 4 Discussion

The development and validation of the AtiPhoS represent a significant advancement in gaining a deeper understanding of the fear and shame resulting from the cultural mechanisms of honor‐based societies. The AtiPhoS demonstrated robust psychometric properties. High internal consistency across the four study phases indicates that the scale effectively captures the essence of atimiaphobia. The test–retest reliability results further reinforce the stability of the construct over time. The factor structure of the AtiPhoS was established through EFA and confirmed via CFA. The EFA results demonstrated adequate sampling adequacy and significant item correlations, leading to a refined 15‐item scale comprising four distinct subscales: (1) fear of being labeled shameless, (2) fear of violating social norms, (3) fear of public judgment, and (4) fear of losing self‐respect and honor. The final model displayed strong factor loadings, reinforcing the theoretical underpinnings of atimiaphobia. The CFA further validated the structure, with satisfactory model fit indices, including CFI, TLI, RMSEA, and SRMR, confirming that the AtiPhoS reliably captures the multidimensional nature of atimiaphobia. The MIRT results provide strong evidence of item quality and scale precision at the latent‐trait level, reinforcing the classical test theory findings and confirming that the AtiPhoS functions effectively as a multidimensional measure of atimiaphobia. Item 12 showed misfit and may benefit from revision in future studies.

The AtiPhoS demonstrated strong convergent validity, as indicated by its significant correlation with the Experience of Shame Scale and the anxiety subscale of the Depression, Anxiety, and Stress Scale. These findings suggest that atimiaphobia shares conceptual similarities with generalized shame experiences and anxiety disorders, reinforcing its legitimacy as a psychological construct. The connection between atimiaphobia, shame, anxiety, and several other psychological problems is justifiable (Brown 2006; Corrigan 2004; Fergus et al. 2010; Treeby et al. 2016; Zhu et al. 2019). Honor and social reputation play a crucial role in shaping individual behaviors and psychological states within honor cultures (Travaglino et al. 2024). These cultures place a high value on maintaining a good reputation and self‐respect (Aslani et al. 2016; Uskul and Cross 2019), which significantly influences how individuals respond to social threats and manage their mental health. Individuals in honor cultures who perceive a threat to their reputation may experience increased depressive symptoms (Foster and Bock 2024). This is particularly true for those with low perceived reputation, as reputation damage can lead to mental health detriments (Foster and Bock 2024). Additionally, concerns about reputation can lead to stigmatization and decreased utilization of mental health services, as seeking help may be seen as damaging to one's social standing (Foster et al. 2021; Husain 2020). The strong inverse predictive validity of AtiPhoS for social intelligence indicates that individuals with heightened atimiaphobia may struggle with adaptive social interactions, possibly due to the pervasive fear of negative judgment and social ostracization (Macovei et al. 2023). Social intelligence refers to the ability to interact effectively with others while demonstrating key practical skills such as understanding social contexts, navigating challenges effectively, recognizing others' emotions and distress, and maintaining personal autonomy and self‐efficacy during social interactions (Albrecht 2005; Husain 2025). Individuals with atimiaphobia may exhibit lower levels of social intelligence due to their heightened sensitivity to social judgment (Magrì 2021), fear of violating cultural norms (Bierbrauer 1992), and preoccupation with maintaining honor and reputation (Bock and Brown 2021).

The current study also identified significant demographic variations in atimiaphobia. Age demonstrated a positive correlation with atimiaphobia and all its sub‐factors, suggesting that as individuals grow older, they become more susceptible to concerns about maintaining honor and adhering to societal expectations. Studies have shown that as people age, they tend to become more conservative, aligning more closely with traditional cultural values and norms (Van Hiel and Brebels 2011). Education did not exhibit a significant correlation with overall atimiaphobia levels; however, it was positively associated with the fear of violating social norms and the fear of public judgment. This finding suggests that individuals with higher education levels may develop a heightened awareness of societal expectations and, consequently, a greater sensitivity to the fear of nonconformity and external judgment. While the direct correlation between higher education and increased sensitivity to fears of nonconformity and external judgment is not established in the current literature, education's role in promoting critical thinking may influence individuals' responses to social conformity pressures. Further empirical research is needed to explore this relationship comprehensively.

Gender differences in atimiaphobia were also highlighted in the current study. Women displayed significantly higher levels of atimiaphobia than men across all sub‐factors. This finding aligns with prior research on gendered socialization patterns in honor cultures, where women often face stricter moral and behavioral expectations than men (Husain, Ijaz, et al. 2025). The heightened fear of being labeled shameless or failing to adhere to social norms reflects the disproportionate burden placed on women to uphold cultural standards of honor and respectability (Husain 2021). Several societies often reinforce female inferiority and impose strict social norms that restrict women's rights (Husain, Tooba, et al. 2024). Women are expected to uphold family honor, maintain an ethical reputation, accept arranged marriages, prioritize their husbands' needs, be attractive, and conform to societal ideals of a “good” woman (Gann‐Bociek and Harvey 2023; Husain 2023; Husain and Aziz 2014). These cultural pressures contribute to increased vulnerability to mental health issues, including atimiaphobia. Marital status further influenced atimiaphobia levels, with married individuals reporting significantly higher atimiaphobia across all sub‐factors compared with their unmarried counterparts. Marriage, particularly in honor cultures, is often accompanied by heightened social scrutiny and expectations regarding moral conduct and familial reputation (Husain, Ijaz, et al. 2024; Husain and Gulzar 2015). These results highlight the intensified psychosocial pressures faced by married individuals in maintaining their perceived honor.

The present study has important implications for psychological research, cross‐cultural studies, and public mental health, particularly within honor cultures and shame societies. By operationalizing fear of losing honor or being labeled shameless as a measurable psychological condition, the AtiPhoS provides researchers with a tool to systematically investigate a form of fear that is culturally salient yet underexamined at the individual psychological level.

From a research perspective, the availability of a dedicated measure enables empirical examination of how honor‐related fear relates to a range of psychosocial outcomes, including chronic stress, emotional suppression, interpersonal vigilance, and maladaptive self‐regulation strategies. Prior literature suggests that sustained fear related to social reputation and moral evaluation may be associated with heightened anxiety, rumination, social withdrawal, and reduced psychological wellbeing. However, without a construct‐specific instrument, such associations have remained diffuse and indirectly inferred. The AtiPhoS allows these relationships to be examined directly, facilitating more precise theorizing about the psychological mechanisms through which honor‐ and shame‐based cultural norms influence individual functioning.

From a public mental health perspective, fear of honor loss may contribute to silent psychological burden in communities where social reputation is tightly regulated and moral transgressions, real or perceived, carry enduring social consequences. Individuals experiencing elevated fear of being labeled shameless may engage in excessive conformity, emotional inhibition, or avoidance of help‐seeking to protect their reputational standing. Such patterns may not reach clinical attention yet can meaningfully impair quality of life, interpersonal relationships, and adaptive coping. Recognizing and measuring this fear therefore has relevance for preventive mental health efforts and culturally responsive psychosocial interventions.

Importantly, the present study does not propose atimiaphobia as a diagnostic category, nor does it claim clinical validation. Instead, it provides a psychometrically sound instrument that can serve as a foundation for future research examining the conditions under which fear of honor loss becomes psychologically burdensome, its interaction with existing mental health constructs, and its potential role in pathways to distress or functional impairment. Future studies employing longitudinal designs, clinical samples, and comparative models will be essential to further clarify these issues.

Future research should continue to examine the applicability and validity of the AtiPhoS across diverse cultural contexts and populations in order to further clarify the construct and its psychological correlates. Future research may further evaluate the psychometric properties of the AtiPhoS using Item Response Theory approaches to examine item discrimination and response category functioning in greater detail.

The current study was conducted within the Pakistani cultural context, which is widely characterized as an honor‐oriented society where family reputation, moral standing, and social evaluation play a central role in shaping individual identity and behavior. In such contexts, concerns related to honor preservation and avoidance of shame may be particularly salient, which could influence how individuals experience and report fear of losing honor or being labeled shameless. As a result, the findings and the psychometric properties of the Atimiaphobia Scale (AtiPhoS) should be interpreted with consideration of this specific sociocultural background.

Because the experience and perception of honor and shame vary across societies, the generalizability of the present findings to other cultural contexts may be limited. Future research should therefore examine the applicability and validity of the AtiPhoS in more diverse populations and cultural environments. In particular, studies conducted in more individualistic cultural settings, where personal autonomy and individual achievement may be more central to social identity than family honor, would help determine whether similar patterns of fear related to reputational judgment emerge or whether the construct manifests differently across cultural contexts.

## 5 Conclusion

The present study contributes to the psychological study of honor cultures by developing and validating the AtiPhoS, a psychometric instrument designed to measure fear of losing honor or being labeled shameless. Drawing on theoretical perspectives related to honor cultures, shame societies, and reputational concerns, the study operationalized this culturally embedded fear as a measurable psychological construct and established its factorial structure and initial validity. The findings provide empirical support for the reliability and construct validity of the AtiPhoS, offering researchers a systematic tool for investigating individual differences in fear related to honor loss and reputational judgment.

By introducing a dedicated measure of this construct, the study opens new avenues for examining how culturally embedded concerns about honor and shame influence psychological experiences, social behavior, and interpersonal functioning. The availability of the AtiPhoS enables future research to more precisely investigate the psychological implications of honor‐related fear within societies where moral reputation and social evaluation play a central role in shaping individual identity and social relationships.

## Funding

The authors have nothing to report.

## Ethics Statement

Ethical approval was granted by the departmental review committee at COMSATS University (Code CUI‐ISB/HUM/ERC‐CPA/2024–028).

## Consent

Informed consent was obtained from the participants. All the procedures performed in this study were in accordance with the 1964 Helsinki Declaration and its later amendments.

## Conflicts of Interest

The authors declare no conflicts of interest.

## Data Availability

The data that support the findings of this study are available on request from the corresponding author. The data are not publicly available due to privacy or ethical restrictions.

## References

[pchj70095-bib-0001] Albrecht, K. 2005. “Social Intelligence: The New Science of Success.” Journal of Chemical Information and Modeling 53, no. 9: 1689–1699.

[pchj70095-bib-0002] Andrews, B. , M. Qian , and J. D. Valentine . 2002. “Predicting Depressive Symptoms With a New Measure of Shame: The Experience of Shame Scale.” British Journal of Clinical Psychology 41, no. 1: 29–42. 10.1348/014466502163778. 11931676

[pchj70095-bib-0003] Aslani, S. , J. Ramirez‐Marin , J. Brett , et al. 2016. “Dignity, Face, and Honor Cultures: A Study of Negotiation Strategy and Outcomes in Three Cultures.” Journal of Organizational Behavior 37, no. 8: 1178–1201. 10.1002/job.2095.

[pchj70095-bib-0004] Asmundson, G. J. G. 2002. “Anxiety and Related Factors in Chronic Pain.” Pain Research and Management 7: 321804. 10.1155/2002/321804. 16231062

[pchj70095-bib-0005] Barrett, N. F. 2015. “A Confucian Theory of Shame.” Sophia 54, no. 2: 143–163. 10.1007/s11841-014-0426-0.

[pchj70095-bib-0006] Bedford, O. A. 2004. “The Individual Experience of Guilt and Shame in Chinese Culture.” Culture & Psychology 10, no. 1: 29–52. 10.1177/1354067X04040929.

[pchj70095-bib-0007] Berkovski, Y. S. 2015. “A Hobbesian Theory of Shame.” Southern Journal of Philosophy 53, no. 2: 125–150. 10.1111/sjp.12101.

[pchj70095-bib-0008] Bhagat, R. S. , and G. Hofstede . 2002. “Culture's Consequences: Comparing Values, Behaviors, Institutions, and Organizations Across Nations.” Academy of Management Review 27, no. 3: 460–462. https://journals.aom.org/doi/10.5465/amr.2002.7389951.

[pchj70095-bib-0009] Bierbrauer, G. 1992. “Reactions to Violation of Normative Standards: A Cross‐Cultural Analysis of Shame and Guilt.” International Journal of Psychology 27, no. 2: 181–193. 10.1080/00207599208246874.

[pchj70095-bib-0010] Blea, J. , D. C. Wang , C. L. Kim , et al. 2021. “The Experience of Financial Well‐Being, Shame, and Mental Health Outcomes in Seminary Students.” Pastoral Psychology 70, no. 4: 299–314. 10.1007/s11089-021-00963-4.

[pchj70095-bib-0011] Bock, J. E. , and R. P. Brown . 2021. “To Be Liked or Feared: Honor‐Oriented Men's Sensitivity to Masculine Reputation Concerns Depends on Status‐Seeking Strategy.” Personality and Individual Differences 173: 110615. 10.1016/j.paid.2020.110615.

[pchj70095-bib-0012] Boiger, M. , B. Mesquita , Y. Uchida , and L. F. Barrett . 2013. “Condoned or Condemned: The Situational Affordance of Anger and Shame in the United States and Japan.” Personality and Social Psychology Bulletin 39, no. 4: 540–553. 10.1177/0146167213478201. 23471319

[pchj70095-bib-0013] Brown, B. 2006. “Shame Resilience Theory: A Grounded Theory Study on Women and Shame.” Families in Society 87, no. 1: 43–52. 10.1606/1044-3894.3483.

[pchj70095-bib-0014] Brown, R. P. , K. Baughman , and M. Carvallo . 2018. “Culture, Masculine Honor, and Violence Toward Women.” Personality and Social Psychology Bulletin 44, no. 4: 538–549. 10.1177/0146167217744195. 29241416

[pchj70095-bib-0015] Buechler, S. 2008. “Shaming Psychoanalytic Candidates.” Psychoanalytic Inquiry 28, no. 3: 361–372. 10.1080/07351690801962430.

[pchj70095-bib-0016] Chalmers, R. P. 2012. “Mirt: A Multidimensional Item Response Theory Package for the R Environment.” Journal of Statistical Software 48, no. 6: 1–29. 10.18637/jss.v048.i06.

[pchj70095-bib-0017] Christianson, M. , Å. Teiler , and C. Eriksson . 2021. ““A Woman's Honor Tumbles Down on All of Us in the Family, but a Man's Honor Is Only His”: Young Women's Experiences of Patriarchal Chastity Norms.” International Journal of Qualitative Studies on Health and Well‐Being 16, no. 1: 1862480. 10.1080/17482631.2020.1862480. 33345754 PMC7751406

[pchj70095-bib-0018] Cihangir, S. 2013. “Gender Specific Honor Codes and Cultural Change.” Group Processes & Intergroup Relations 16, no. 3: 319–333. 10.1177/1368430212463453.

[pchj70095-bib-0019] Clara, I. P. , B. J. Cox , and M. W. Enns . 2001. “Confirmatory Factor Analysis of the Depression‐Anxiety‐Stress Scales in Depressed and Anxious Patients.” Journal of Psychopathology and Behavioral Assessment 23, no. 1: 61–67. 10.1023/A:1011095624717.

[pchj70095-bib-0020] Cohen, J. 2013. “Statistical Power Analysis for the Behavioral Sciences.” In Statistical Power Analysis for the Behavioral Sciences, 2nd ed. Routledge. 10.4324/9780203771587.

[pchj70095-bib-0021] Comrey, A. L. , and H. B. Lee . 1992. A First Course in Factor Analysis. 2nd ed. Taylor & Francis Group. 10.4324/9781315827506.

[pchj70095-bib-0022] Corrigan, P. 2004. “How Stigma Interferes With Mental Health Care.” American Psychologist 59, no. 7: 614–625. 10.1037/0003-066X.59.7.614. 15491256

[pchj70095-bib-0023] Crawford, J. R. , and J. D. Henry . 2003. “The Depression Anxiety Stress Scales (DASS): Normative Data and Latent Structure in a Large Non‐Clinical Sample.” British Journal of Clinical Psychology 42, no. 2: 111–131. 10.1348/014466503321903544. 12828802

[pchj70095-bib-0024] Cross, S. E. , A. K. Uskul , B. Gerçek‐Swing , C. Alözkan , and B. Ataca . 2013. “Confrontation Versus Withdrawal: Cultural Differences in Responses to Threats to Honor.” Group Processes & Intergroup Relations 16, no. 3: 345–362. 10.1177/1368430212461962.

[pchj70095-bib-0025] Cross, S. E. , A. K. Uskul , B. Gerçek‐Swing , et al. 2014. “Cultural Prototypes and Dimensions of Honor.” Personality and Social Psychology Bulletin 40, no. 2: 232–249. 10.1177/0146167213510323. 24311437

[pchj70095-bib-0026] Dayan, H. 2021. “Female Honor Killing: The Role of Low Socio‐Economic Status and Rapid Modernization.” Journal of Interpersonal Violence 36, no. 19–20: NP10393–NP10410. 10.1177/0886260519872984. 31524058

[pchj70095-bib-0027] de Hooge, I. E. , M. Zeelenberg , and S. M. Breugelmans . 2010. “Restore and Protect Motivations Following Shame.” Cognition and Emotion 24, no. 1: 111–127. 10.1080/02699930802584466.

[pchj70095-bib-0028] Dearing, R. L. , J. P. E. Tangney , and M. Sweezy . 2011. “Shame in the Therapy Hour.” In American Journal of Psychotherapy, edited by R. L. Dearing and J. P. Tangney , vol. 65, 393–394. American Psychological Association. 10.1176/appi.psychotherapy.2011.65.4.393.

[pchj70095-bib-0029] D'Lima, T. , J. L. Solotaroff , and R. P. Pande . 2020. “For the Sake of Family and Tradition: Honour Killings in India and Pakistan.” ANTYAJAA: Indian Journal of Women and Social Change 5, no. 1: 22–39. 10.1177/2455632719880852.

[pchj70095-bib-0030] Dunaetz, D. R. 2021. “Approaching Honor and Shame With Humility: Limitations to Our Current Understanding.” Missiology: An International Review 49, no. 4: 402–416. 10.1177/0091829621995531.

[pchj70095-bib-0031] Fergus, T. A. , D. P. Valentiner , P. B. McGrath , and S. Jencius . 2010. “Shame‐ and Guilt‐Proneness: Relationships With Anxiety Disorder Symptoms in a Clinical Sample.” Journal of Anxiety Disorders 24, no. 8: 811–815. 10.1016/j.janxdis.2010.06.002. 20591613

[pchj70095-bib-0032] Foster, S. , and J. Bock . 2024. “Perceived Reputation Moderates the Link Between Honor Concerns and Depressive Symptoms.” Journal of Social Psychology 165, no. 6: 769–778. 10.1080/00224545.2024.2334036. 38530884

[pchj70095-bib-0033] Foster, S. , M. Carvallo , J. Lee , and I. Bernier . 2021. “Honor and Seeking Mental Health Services: The Roles of Stigma and Reputation Concerns.” Journal of Cross‐Cultural Psychology 52, no. 2: 178–183. 10.1177/0022022120982070.

[pchj70095-bib-0034] Fromson, P. M. 2006. “Evoking Shame and Guilt: A Comparison of Two Theories.” Psychological Reports 98, no. 1: 99–105. 10.2466/PR0.98.1.99-105. 16673958

[pchj70095-bib-0035] Furukawa, E. , J. Tangney , and F. Higashibara . 2012. “Cross‐Cultural Continuities and Discontinuities in Shame, Guilt, and Pride: A Study of Children Residing in Japan, Korea and the USA.” Self and Identity 11, no. 1: 90–113. 10.1080/15298868.2010.512748.

[pchj70095-bib-0036] Gann‐Bociek, M. , and R. D. Harvey . 2023. “Gender Stereotypes.” Stereotypes: The Incidence and Impacts of Bias, edited by J. T. Nadler and E. C. Voyles , 175–193. Praeger. 10.5040/9798216018902.ch-010.

[pchj70095-bib-0037] Gengler, J. J. , M. F. Alkazemi , and A. Alsharekh . 2021. “Who Supports Honor‐Based Violence in the Middle East? Findings From a National Survey of Kuwait.” Journal of Interpersonal Violence 36, no. 11–12: NP6013–NP6039. 10.1177/0886260518812067. 30449232

[pchj70095-bib-0038] Husain, W. 2020. “Barriers in Seeking Psychological Help: Public Perception in Pakistan.” Community Mental Health Journal 56, no. 1: 75–78. 10.1007/s10597-019-00464-y. 31542848

[pchj70095-bib-0039] Husain, W. 2021. “Women Are the Better Halves: Gender‐Based Variations in Virtues and Character Strengths.” Journal of Human Values 28, no. 2: 103–114. 10.1177/09716858211039984.

[pchj70095-bib-0040] Husain, W. 2023. “Charismaphobia: Diagnosis and Measurement of the Psychodermatological Symptoms.” Journal of Skin and Stem Cell 10, no. 2: e137387. 10.5812/jssc-137387.

[pchj70095-bib-0041] Husain, W. 2025. “Atimiaphobia: A Phenomenological Account of the Fear of Losing Honor or Being Labeled Shameless due to the Sexual Values Assigned to Femininity.” OBM Integrative and Complementary Medicine 10, no. 3: 1–30. 10.21926/obm.icm.2503037.

[pchj70095-bib-0042] Husain, W. , and N. Aziz . 2014. “The Levels of Body Esteem Among Veiled and Unveiled Women.” FWU Journal of Social Sciences 8, no. 1: 46–49.

[pchj70095-bib-0043] Husain, W. , and A. Gulzar . 2015. “The Psychosocial Preferences in Mate Selection Among Pakistanis.” FWU Journal of Social Sciences 9, no. 1: 29–31.

[pchj70095-bib-0044] Husain, W. , and A. Gulzar . 2020. “Translation, Adaptation and Validation of Depression, Anxiety and Stress Scale in Urdu.” Insights on the Depression and Anxiety 4, no. 1: 1–4. 10.29328/journal.ida.1001011.

[pchj70095-bib-0045] Husain, W. , F. Ijaz , M. A. Husain , A. Ammar , K. Trabelsi , and H. Jahrami . 2025. “Development and Validation of the Women Autonomy Scale for Measuring Psychosocial Freedom From Conventional Gender Roles.” BMC Psychology 13: 88. 10.1186/s40359-025-02393-w. 39885560 PMC11783771

[pchj70095-bib-0046] Husain, W. , F. Ijaz , M. A. Husain , M. Zulfiqar , and J. Khalique . 2024. “Love, Sex, Respect, and Physical Attractiveness in Marital Satisfaction and Remarriage: A Comparison Between Monogamous and Polygynous Marriages. *Interpersona: An International Journal on* .” Personal Relationships 18: 174–188. 10.5964/ijpr.11759.

[pchj70095-bib-0047] Husain, W. , A. Kamal , F. Ijaz , et al. 2025. “Redefining Social Intelligence Through Self‐Efficacy and Personal Autonomy: Development and Validation of the Efficient Social Intelligence Scale.” Journal of Human Behavior in the Social Environment 1–19: 1–19. 10.1080/10911359.2025.2454265.

[pchj70095-bib-0048] Husain, W. , N. Tooba , M. A. Husain , and F. Ijaz . 2024. “The Perception of Feminism and the Inclination for Empowerment Among Educated Muslim Women From Pakistan.” Middle East Journal of Islamic Studies and Culture 4, no. 1: 11–19. 10.36348/mejisc.2024.v04i01.003.

[pchj70095-bib-0049] Kaya, N. , and N. Turan . 2018. “Attitudes Toward Honor and Violence Against Women for Honor in the Context of the Concept of Privacy: A Study of Students in the Faculty of Health Sciences.” Connectist: Istanbul University Journal of Communication Sciences 54: 65–84. 10.26650/connectist433995.

[pchj70095-bib-0050] Kenardy, J. , L. Evans , and T. P. S. Oei . 1992. “The Latent Structure of Anxiety Symptoms in Anxiety Disorders.” American Journal of Psychiatry 149, no. 8: 1058–1061. 10.1176/ajp.149.8.1058. 1636806

[pchj70095-bib-0051] Kline, R. B. 2023. Principles and Practice of Structural Equation Modeling. Guilford Publications.

[pchj70095-bib-0052] Kõlves, K. , N. Ide , and D. De Leo . 2011. “Marital Breakdown, Shame, and Suicidality in Men: A Direct Link?” Suicide and Life‐Threatening Behavior 41, no. 2: 149–159. 10.1111/j.1943-278X.2011.00021.x. 21470294

[pchj70095-bib-0053] Kotrotsiou, S. , M. I. Gouva , K. Gourgoulianis , T. Paralikas , C. Hatzoglou , and N. Skenteris . 2013. “1615 – Internal Shame: Differences Between Men and Women Greek Roma.” European Psychiatry 28: 1. 10.1016/s0924-9338(13)76612-4.21920709

[pchj70095-bib-0054] Leung, A. K. Y. , and D. Cohen . 2011. “Within‐ and Between‐Culture Variation: Individual Differences and the Cultural Logics of Honor, Face, and Dignity Cultures.” Journal of Personality and Social Psychology 100, no. 3: 507–526. 10.1037/a0022151. 21244179

[pchj70095-bib-0055] Lopez‐Zafra, E. , N. Rodríguez‐Espartal , and M. M. Ramos‐Alvarez . 2020. “Women's and Men's Role in Culture of Honor Endorsement Within Families.” European Journal of Women's Studies 27, no. 1: 72–88. 10.1177/1350506818824369.

[pchj70095-bib-0056] Lovibond, P. F. , and S. H. Lovibond . 1995. “The Structure of Negative Emotional States: Comparison of the Depression Anxiety Stress Scales (DASS) With the Beck Depression and Anxiety Inventories.” Behaviour Research and Therapy 33, no. 3: 335–343. 10.1016/0005-7967(94)00075-U. 7726811

[pchj70095-bib-0057] Macovei, C. M. , Ș. Bumbuc , and F. Martinescu‐Bădălan . 2023. “The Role of Personality Traits in Mediating the Relation Between Fear of Negative Evaluation and Social Interaction Anxiety.” Frontiers in Psychology 14: 1268052. 10.3389/fpsyg.2023.1268052. 37928579 PMC10621049

[pchj70095-bib-0058] Magrì, E. 2021. “Towards a Phenomenological Account of Social Sensitivity.” Phenomenology and the Cognitive Sciences 20, no. 4: 635–653. 10.1007/s11097-020-09689-9.

[pchj70095-bib-0059] Mayer, J. D. , K. G. Phillips , and A. Barry . 2015. “Getting the Message: The Adaptive Potential of Interpersonal Judgments.” Review of General Psychology 19, no. 1: 39–51. 10.1037/gpr0000025.

[pchj70095-bib-0060] Mills, E. , and S. Kellington . 2012. “Using Group Art Therapy to Address the Shame and Silencing Surrounding Children's Experiences of Witnessing Domestic Violence.” International Journal of Art Therapy: Inscape 17, no. 1: 3–12. 10.1080/17454832.2011.639788.

[pchj70095-bib-0061] Mosquera, P. M. R. , A. Fischer , A. Manstead , and R. Zaalberg . 2008. “Attack, Disapproval, or Withdrawal? The Role of Honour in Anger and Shame Responses to Being Insulted.” Cognition and Emotion 22, no. 8: 1471–1498. 10.1080/02699930701822272.

[pchj70095-bib-0062] Nash, S. 2017. “On Researching Shame in the Church.” Practical Theology 10, no. 4: 396–406. 10.1080/1756073X.2017.1374791.

[pchj70095-bib-0063] Nowak, A. , M. J. Gelfand , W. Borkowski , D. Cohen , and I. Hernandez . 2016. “The Evolutionary Basis of Honor Cultures.” Psychological Science 27, no. 1: 12–24. 10.1177/0956797615602860. 26607976

[pchj70095-bib-0064] Olsthoorn, P. 2005. “Honor as a Motive for Making Sacrifices.” Journal of Military Ethics 4, no. 3: 183–197. 10.1080/15027570500230262.

[pchj70095-bib-0065] Orth, U. , R. W. Robins , and C. J. Soto . 2010. “Tracking the Trajectory of Shame, Guilt, and Pride Across the Life Span.” Journal of Personality and Social Psychology 99, no. 6: 1061–1071. 10.1037/a0021342. 21114354

[pchj70095-bib-0066] Osborne, J. W. , A. B. Costello , and J. T. Kellow . 2008. “Best Practices in Exploratory Factor Analysis.” In Best Practices in Quantitative Methods, 86–99. SAGE Publications, Inc.

[pchj70095-bib-0067] Panayiotou, G. , M. Karekla , D. Georgiou , E. Constantinou , and M. Paraskeva‐Siamata . 2017. “Psychophysiological and Self‐Reported Reactivity Associated With Social Anxiety and Public Speaking Fear Symptoms: Effects of Fear Versus Distress.” Psychiatry Research 255: 278–286. 10.1016/j.psychres.2017.05.044. 28599192

[pchj70095-bib-0068] Patterson, C. 2019. “The World of Honor and Shame in the New Testament: Alien or Familiar?” Biblical Theology Bulletin 49, no. 1: 4–14. 10.1177/0146107918818038.

[pchj70095-bib-0069] Richardson, T. , P. Elliott , R. Roberts , and M. Jansen . 2017. “A Longitudinal Study of Financial Difficulties and Mental Health in a National Sample of British Undergraduate Students.” Community Mental Health Journal 53, no. 3: 344–352. 10.1007/s10597-016-0052-0. 27473685 PMC5337246

[pchj70095-bib-0070] Robertson, T. E. , D. Sznycer , A. W. Delton , J. Tooby , and L. Cosmides . 2018. “The True Trigger of Shame: Social Devaluation Is Sufficient, Wrongdoing Is Unnecessary.” Evolution and Human Behavior 39, no. 5: 566–573. 10.1016/j.evolhumbehav.2018.05.010.

[pchj70095-bib-0071] Rodriguez Mosquera, P. M. 2016. “On the Importance of Family, Morality, Masculine, and Feminine Honor for Theory and Research.” Social and Personality Psychology Compass 10, no. 8: 431–442. 10.1111/spc3.12262.

[pchj70095-bib-0072] Rudolph, K. D. , and C. S. Conley . 2005. “The Socioemotionol Costs and Benefits of Social‐Evaluative Concerns: Do Girls Care Too Much?” Journal of Personality 73, no. 1: 115–138. 10.1111/j.1467-6494.2004.00306.x. 15660675 PMC3158587

[pchj70095-bib-0073] Sheikh, S. , and R. Janoff‐Bulman . 2010. “The “Shoulds” and “Should Nots” of Moral Emotions: A Self‐Regulatory Perspective on Shame and Guilt.” Personality and Social Psychology Bulletin 36, no. 2: 213–224. 10.1177/0146167209356788. 20008966

[pchj70095-bib-0074] Shier, A. , and E. Shor . 2016. ““Shades of Foreign Evil”: “Honor Killings” and “Family Murders” in the Canadian Press.” Violence Against Women 22, no. 10: 1163–1188. 10.1177/1077801215621176. 26712236

[pchj70095-bib-0075] Sznycer, D. , L. Cosmides , and J. Tooby . 2017. “Adaptationism Carves Emotions at Their Functional Joints.” Psychological Inquiry 28, no. 1: 56–62. 10.1080/1047840X.2017.1256132.

[pchj70095-bib-0076] Sznycer, D. , E. Schniter , J. Tooby , and L. Cosmides . 2015. “Regulatory Adaptations for Delivering Information: The Case of Confession.” Evolution and Human Behavior 36, no. 1: 44–51. 10.1016/j.evolhumbehav.2014.08.008. PMC4313746 25663798

[pchj70095-bib-0077] Tabachnick, B. G. , L. S. Fidell , and J. B. Ullman . 2013. Using Multivariate Statistics. 6th ed. Pearson.

[pchj70095-bib-0078] Tangney, J. P. 2004. “Perfectionism and the Self‐Conscious Emotions: Shame, Guilt, Embarrassment, and Pride.” In Perfectionism: Theory, Research, and Treatment, edited by T. Dalgleish and M. J. Power , 199–215. John Wiley & Sons, Ltd. 10.1037/10458-008.

[pchj70095-bib-0079] Tangney, J. P. , J. Stuewig , and A. G. Martinez . 2014. “Two Faces of Shame: The Roles of Shame and Guilt in Predicting Recidivism.” Psychological Science 25, no. 3: 799–805. 10.1177/0956797613508790. 24395738 PMC4105017

[pchj70095-bib-0080] Thomas, R. , R. Deighton , M. Mizuno , and S. Y. Fujii . 2020. “Shame and Self‐Conscious Emotions in Japan and Australia: Evidence for a Third Shame Logic.” Culture & Psychology 26, no. 3: 622–638. 10.1177/1354067X19851024.

[pchj70095-bib-0081] Travaglino, G. A. , M. T. Friehs , P. F. Kotzur , and D. Abrams . 2024. “Honor Values as Identity Content: Evidence From a Three‐Wave Longitudinal Study.” Journal of Cross‐Cultural Psychology 55, no. 3: 278–291. 10.1177/00220221241230959. 38496723 PMC10942845

[pchj70095-bib-0082] Treeby, M. S. , C. Prado , S. M. Rice , and S. F. Crowe . 2016. “Shame, Guilt, and Facial Emotion Processing: Initial Evidence for a Positive Relationship Between Guilt‐Proneness and Facial Emotion Recognition Ability.” Cognition and Emotion 30, no. 8: 1504–1511. 10.1080/02699931.2015.1072497. 26264817

[pchj70095-bib-0083] Uskul, A. K. , and S. E. Cross . 2019. “The Social and Cultural Psychology of Honour: What Have We Learned From Researching Honour in Turkey?” European Review of Social Psychology 30, no. 1: 39–73. 10.1080/10463283.2018.1542903.

[pchj70095-bib-0084] Van Hiel, A. , and L. Brebels . 2011. “Conservatism Is Good for You: Cultural Conservatism Protects Self‐Esteem in Older Adults.” Personality and Individual Differences 50, no. 1: 120–123. 10.1016/j.paid.2010.09.002.

[pchj70095-bib-0085] van Osch, Y. , S. M. Breugelmans , M. Zeelenberg , and P. Bölük . 2013. “A Different Kind of Honor Culture: Family Honor and Aggression in Turks.” Group Processes & Intergroup Relations 16, no. 3: 334–344. 10.1177/1368430212467475.

[pchj70095-bib-0086] Vizin, G. , R. Urbán , and Z. Unoka . 2016. “Shame, Trauma, Temperament and Psychopathology: Construct Validity of the Experience of Shame Scale.” Psychiatry Research 246: 62–69. 10.1016/j.psychres.2016.09.017. 27664547

[pchj70095-bib-0087] Wong, Y. , and J. Tsai . 2007. “Cultural Models of Shame and Guilt.” In The Self‐Conscious Emotions: Theory and Research, edited by J. L. Tracy , R. W. Robins , and J. P. Tangney , 209–223. The Guilford Press.

[pchj70095-bib-0088] Yakeley, J. 2018. “Shame, Culture and Mental Health.” Nordic Journal of Psychiatry 72, no. sup1: S20–S22. 10.1080/08039488.2018.1525641. 30489215

[pchj70095-bib-0089] Zhu, R. , Z. Xu , H. Tang , et al. 2019. “The Effect of Shame on Anger at Others: Awareness of the Emotion‐Causing Events Matters.” Cognition and Emotion 33, no. 4: 696–708. 10.1080/02699931.2018.1489782. 29932822

